# P02-004 - AIDs in a registry of children in North America

**DOI:** 10.1186/1546-0096-11-S1-A111

**Published:** 2013-11-08

**Authors:** JS Hausmann, CM Biggs, D Goldsmith, F Dedeoglu

**Affiliations:** 1 Program in Rheumatology, Division of Immunology, Boston Children's Hospital, Boston, MA, USA; 2 Section of Rheumatology, St Christopher's Hospital for Children, Drexel University College of Medicine, Philadelphia, PA, USA

## Introduction

Autoinflammatory diseases (AIDs) are characterized by recurrent episodes of systemic and organ-specific inflammation. If unrecognized and untreated, they may cause significant morbidity and mortality.

Our understanding and management of these disorders has improved markedly over the last decade. Nevertheless, the majority of children with periodic fevers do not have mutations in known periodic fever syndrome genes. Even in patients with known mutations, the clinical phenotype may vary greatly, likely as a result of the environment. Given the rarity of AIDs, registries of patients with these disorders have been established in some countries.

The Childhood Arthritis and Rheumatology Research Alliance (CARRA) Registry is a multicenter, observational database of North American children with rheumatic diseases, including those with AIDs. Patients with defined rheumatic diseases who are 21 years of age or younger and are seen at a CARRA site are eligible to enroll. This study is the first report of patients with AIDs within this registry.

## Objectives

To describe the demographic data of patients with AIDs in a North American registry of rheumatic disorders.

## Methods

We conducted a cross-sectional study of children and adolescents with AIDs enrolled in the CARRA Registry. Enrollment in the database began in May 2011 and our study included data as of March 1, 2013. Inclusion criteria included diagnoses of specific monogenic and polygenic AIDs, as well as suspected AIDs with undefined diagnoses. Baseline data at enrollment is reported and analyzed.

## Results

As of March 1, 2013, 7,931 patients were enrolled in the CARRA database. Fifty-one (0.64%) were diagnosed with AIDs, ranging in ages at enrollment from 2.5 to 20.7 years. There were 28 females (54.9%). Thirty-nine (76.5%) patients were White, 6 (11.8%) were Latino, and there were 2 (3.9%) patients from each of the following ethnic groups: American Indian/Alaskan Native, Black/African American, and Middle Eastern.

Of the 51 patients with AIDs, 20 (39.2%) had CRMO, 7 (13.7%) had PFAPA, 7 (13.7%) had FMF, 4 (7.8%) had TRAPS, 2 (3.9%) had MWS, and 1 (2%) patient each had PAPA, SAPHO, and NOMID. One (2%) patient had both FMF and TRAPS, and 7 (13.7%) patients had undefined AIDs.

Fifteen (29.4%) patients had genetically confirmed disease. The average length of time between the onset of symptoms and the evaluation by a rheumatologist was 1.35 years, ranging from 0.06 to 5 years. At enrollment, 41 (80.4%) patients had an ACR global functional status of Class I.

The age of onset of each disease varied markedly. NOMID was diagnosed at birth, while SAPHO had the latest average age of diagnosis at 13. Average CHAQ scores ranged from 0 in patients with PAPA and FMF/TRAPS, to 2.38 in the patient with NOMID. Physician global score also ranged from 0 for patients with FMF, PAPA, and FMF/TRAPS, to 8 for the patient with NOMID.

## Conclusion

This study is the first to examine patients with AIDs in a North American, multiethnic registry. Continued observation of these patients and enrollment of new patients should provide meaningful data on these rare diseases in order to facilitate diagnosis, optimize treatment, and improve prognosis. We hope that this effort will encourage further international collaboration to create a truly international registry.

## Disclosure of interest

None declared.

